# Supplementary material for: Research ethics review during the COVID-19 pandemic: An international study
Source: PLoS One. 2024 Apr 16;19(4):e0292512. doi: 10.1371/journal.pone.0292512 (PMC11020390; doi:10.1371/journal.pone.0292512)
Supplement: S1 Appendix — (DOCX) [file pone.0292512.s001.docx]

**Research ethics review during the COVID-19 pandemic: An international study**

Supporting Information

S1 Appendix. Qualtrics questionnaire

Start of Block: Letter of Information and Consent

**INTRODUCTION**

**LETTER OF INFORMATION AND CONSENT**

**Project Title**

Research ethics review during the COVID-19 pandemic: An international study

**Principal Investigators and Contact Information**

Professor Maxwell Smith (Principal Investigator)

Faculty of Health Sciences, School of Health Studies, Western University

[maxwell.smith@uwo.ca](mailto:maxwell.smith@uwo.ca)

519 661-2111 x88589

Professor Ross E.G. Upshur (Co-Principal Investigator)

Dalla Lana School of Public Health, University of Toronto

[ross.upshur@utoronto.ca](mailto:ross.upshur@utoronto.ca)

416-978-6459

**Co-Investigators**

- Dr. Fabio Salamanca-Buentello (Project Coordinator), Lunenfeld – Tanenbaum Research Institute, Sinai Health System, and School of Health Studies, Faculty of Health Sciences, Western University
- Professor Diego Silva, Faculty of Medicine and Health, University of Sydney
- Dr. Kerry Ellen O’Neill, School of Health Studies, Faculty of Health Sciences, and Rotman Institute of Philosophy, Western University
- Dr. Michael Montess, School of Health Studies, Faculty of Health Sciences, and Rotman Institute of Philosophy, Western University
- Dr. Ana Komparic, Public Health Agency of Canada
- Ms. Rachel Katz, Institute for the History and Philosophy of Science and Technology, University of Toronto, and School of Health Studies, Faculty of Health Sciences, Western University

**Sponsor**

This project is funded by a Canadian Institutes of Health Research grant (#C150-2019-11).

**Invitation to Participate**

You are being invited to participate in this survey because of your expertise on the study topic and your membership in an organization providing research ethics approval (such as an ethics review committee, research ethics board, or institutional review board) that reviewed COVID-19-related research during the COVID-19 pandemic.

Our study aims to understand whether ethics review committees worldwide did or did not modify existing procedures, or designed and implemented innovations or new processes, to continue operating during the COVID-19 pandemic, and, if they did, how they accomplished this goal. We also endeavour to identify the kind of support they did or did not receive to help them in this task. Your participation will be invaluable.

Before agreeing to participate in this study, it is important that you read and understand the following information.

**Introduction and Background**

Ethics review committees have faced daunting challenges during the COVID-19 pandemic arising from increased pressure, taxing time constraints, altered priorities, and overwhelming logistical hurdles. Such obstacles have been markedly burdensome in contexts beset with fragile health systems, poor infrastructure, and little experience of medical research. Failure to conduct rapid but in-depth and high-quality reviews could have placed lives at risk and could have resulted in lost opportunities to gain critical knowledge. The COVID-19 pandemic forced ethics review committees to make reasonable adjustments and design innovative strategies to ensure rapid but thorough reviews of research protocols.

**Goals of the Study**

We aim to identify the changes (if any) that your ethics review committee implemented to adapt to the pandemic, particularly in terms of modifying existing procedures; designing and implementing new processes; diversifying membership, especially in terms of inviting external experts; accelerating research protocol review; securing emergency support (including funding); and strengthening local and international collaboration with other ethics review committees.

We also seek to determine whether the success or failure of any changes was evaluated, and whether you believe any modifications should become permanent to everyday functioning of your ethics review committee. The results of our study, which we expect will have considerable international impact, will be widely distributed to key decision-makers worldwide.

**Study Design**

You will participate in a brief, anonymous, international, cross-sectional survey administered using the Qualtrics platform. We are inviting chairs / members of ethics review committees, research ethics boards, and institutional review boards affiliated with diverse types of institutions and organizations (such as academic institutions, government, health care, industry, industry, and non-governmental organizations) from countries in each World Health Organization (WHO) region who have participated in the review of research protocols related to COVID-19. We will balance the group of experts in terms of representation according to sex, type of country (high-income / low- and middle-income), area or expertise, and WHO region.

We will exclude individuals who do not fulfill at least one of the inclusion criteria, who do not submit the survey (that is, who do not reach the end of the questionnaire), or who explicitly request to be withdrawn from the survey.

Quantitative data resulting from the survey will be examined using conventional descriptive statistical analysis. Qualitative data (text responses from open-ended questions) will be evaluated using thematic analysis and the constant comparative method.

**Study Procedures**

Your participation in our study will consist of completing a brief online questionnaire of multiple-choice questions through the Qualtrics platform. This activity will take approximately 15 minutes of your time. You will also be asked basic demographic information that will contribute to frame and contextualize our results.

**Rights of Participants**

Your participation in this study is voluntary. You may decide not to be in this study. Even if you consent to participate, you have the right to not answer individual questions or to withdraw from the study at any time. Choosing not to participate or to leave the study at any time will have no effect on you, your employment status, or your academic standing. Your decision to participate in our study is strictly confidential.

You do not waive any legal right by consenting to this study.

You can skip questions in the survey should you choose not to answer them. You can stop answering the questionnaire at any time.

Please note that participation will not confer authorship on resulting manuscripts.

**Withdrawal from Study**

If you decide to withdraw from the study, you may do so at any time by exiting the survey window. Because we are not collecting any personal identifiers that could link responses to you, it will not be possible to withdraw your data after it is submitted.

**Risks and Harms of Participating in this Study**

Participating in this survey carries no known or anticipated physical, psychological, emotional, social, or legal risks to you.

**Benefits of Participating in this Study**

Our results will provide key insights into the role and functioning of ethics review committees during the COVID-19 pandemic. We expect to assess the success and failure of the strategies designed and implemented by ethics review committees globally to continue providing ethics review during the pandemic. In the long-term, our results may help inform the approaches that ethics review committees should take during future public health emergencies to ensure reviews are provided rapidly and thoroughly despite having to confront daunting challenges.

**Privacy and Confidentiality**

All the information you provide will be kept confidential. The collated responses will not include any of your personal or institutional data. No identifying information will be revealed in subsequent publications of the survey findings or in the raw data deposited in database repositories. Demographic information such as your sex and age range will contribute to frame and contextualize the analysis of our results, but cannot be linked back to you.

We will use the Qualtrics platform for data collection, storage, and analysis. Data will be stored physically in Ireland in Qualtrics servers. While data breaches are possible, they are unlikely to occur. Qualtrics’ privacy policies can be found here:

- <https://www.qualtrics.com/support/survey-platform/getting-started/data-protection-privacy/>
- <https://www.qualtrics.com/uk/platform/gdpr/>

All data will be encrypted in compliance with the Ontario Freedom of Information and Protection of Privacy Act. De-identified study related records may be electronically transmitted outside of Western University and/or its affiliate institutions given that some members of the study team work remotely / from home; study data will be shared with collaborators / researchers external to Western University; and study data may need to be deposited in open access data repositories as a requirement for publication in scientific journals (see below).

All identifiable information will be deleted from the dataset collected to protect the anonymity of individual participants. The de-identified data will be accessible by the study investigators as well as the broader scientific community. More specifically, as a requirement for publication, the data may need to be deposited in a specific data repository or be made otherwise available to other researchers so that it may be inspected and analyzed. The data that will be shared will not contain any information that can identify you.

Data generated from our survey will be kept by the Principal Investigator in a secure location for a minimum of seven years after closure of the study in accordance with Western University’s Faculty Collective Agreement, after which time it will be destroyed. We will ensure that study data are removed from the Qualtrics platform as soon as the project reaches its conclusion.

Delegated institutional representatives of Western University and its Non-Medical Research Ethics Board may require access to your study-related records to monitor the conduct of the research in accordance with regulatory requirements.

**Compensation**

Please be aware that you will not be receiving any compensation for participating in this study.

**Conflict of Interest**

The members of our research team declare that they have no conflict of interest and will receive no financial benefits such as remuneration, intellectual property rights, consultancies, board membership, rights of employment, share ownership, or stock options from any source. Our study does not receive any sponsorship or funding from pharmaceutical companies or any other extra-academic sources.

**Questions About the Study**

If you have questions about this research study, please contact:

Professor Maxwell Smith (Principal Investigator)

School of Health Studies and Rotman Institute of Philosophy, Western University

[maxwell.smith@uwo.ca](mailto:maxwell.smith@uwo.ca)

519 661-2111 x88589

If you have any questions about your rights as a research participant or the conduct of this study, you may contact The Office of Human Research Ethics (519) 661-3036, 1-844-720-9816, email: [ethics@uwo.ca](mailto:ethics@uwo.ca) . This office oversees the ethical conduct of research studies and is not part of the study team. Everything that you discuss will be kept confidential.

**Consent**

Please note that, by answering our research questions, you will be providing implicit consent to participate in our survey. Therefore, submitting the survey is indication of your consent to participate.

**[Please click on the following link if you wish to download a PDF version of the Letter of Information and Consent. This letter is yours to keep for future reference:** [Smith 2022 rore survey letterofinformationandconsent](https://uwo.eu.qualtrics.com/CP/File.php?F=F_6Av32yeToYVnmoC) **]**

**I CONSENT TO PARTICIPATE IN THIS SURVEY**

End of Block: Letter of Information and Consent

Start of Block: CONFIRMATION OF ELIGIBILITY

A

Were you involved in the formal ethics review of **COVID-19-related research*** as a chair, administrator, secretariat, or member of an ethics review committee (for example, a research ethics board, research ethics committee, or institutional review board) during the COVID-19 pandemic? *(*For the purposes of this survey, we define "COVID-19-related research" as studies conducted after March 2020 that relate to SARS-CoV-2, COVID-19, the measures used to diagnose, treat, or prevent COVID-19, or the repercussions of this pandemic or of the preventive, diagnostic, and therapeutic approaches designed and implemented to control it).*

- Yes (1)
- No (2)

| **MESSAGE TO RESPONDENTS WHO ANSWERED “NO” TO QUESTION A:**  **Thank you for your interest in our survey. Unfortunately, we are only seeking responses from those individuals involved in the formal ethics review of COVID-19-related research as chairs, administrators, secretariats, or members of an ethics review committee during the COVID-19 pandemic.**  **However, we do hope there will be other opportunities for future academic and professional interaction.** |
| --- |

End of Block: CONFIRMATION OF ELIGIBILITY

Start of Block: EARLY EXIT

Start of Block: DEMOGRAPHIC AND BACKGROUND INFORMATION

B How do you describe yourself?

- Female (1)
- Male (2)
- Non-binary, gender fluid, and/or two spirit (3)
- Prefer to self-describe (4) ________________________________________________
- Prefer not to disclose (5)

| Page Break |  |
| --- | --- |

C What is your age?

- 18 - 24 years old (1)
- 25 - 34 years old (2)
- 35 - 44 years old (3)
- 45 - 54 years old (4)
- 55 - 64 years old (5)
- 65+ years old (6)
- Prefer not to disclose (7)

| Page Break |  |
| --- | --- |

| 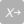 |
| --- |

D In which country do you currently reside (please choose from the list below?

▼ Afghanistan (1) ... Zimbabwe (1357)

End of Block: DEMOGRAPHIC AND BACKGROUND INFORMATION

Start of Block: EXPERIENCE WITH ETHICS REVIEWS

E During the COVID-19 pandemic, how many ethics review committees that reviewed COVID-19-related research were you involved with?

- 1 (1)
- 2 (2)
- 3+ (3)

Skip To: G1 If During the COVID-19 pandemic, how many ethics review committees that reviewed COVID-19-related re... = 1

F Did your time involved with multiple ethics review committees overlap (i.e., were you simultaneously involved in ethics review for multiple committees)?

- Yes (1)
- No (2)

| Page Break |  |
| --- | --- |

NOTE ***For the remainder of the survey, you will be asked questions about your experience with research ethics review during the COVID-19 pandemic. If you had experience with multiple ethics review committees, we ask that you please answer the following questions only in terms of your experience with one committee (e.g., the one you were most involved with).***

| Page Break |  |
| --- | --- |

G1 From the list below, please select the World Health Organization region from which the research protocols reviewed by your ethics committee predominantly originated (select all that apply):

- Africa (1)
- Americas (2)
- Eastern Mediterranean (3)
- Europe (4)
- South-East Asia (5)
- Western Pacific (6)
- Only reviewed research protocols from own country (please specify which country in the text box below): (7) ________________________________________________

| Page Break |  |
| --- | --- |

H For which kind of organization did you provide ethics review of research protocols? (If your ethics committee provides ethics review for multiple organizations, select all that apply.)

- National body (e.g., national ethics committee; national public health organization) (1)
- Sub-national governmental body (2)
- Health care facility (e.g., hospital) (3)
- Academic institution or research institute (e.g., university) (4)
- Private industry (5)
- Non-governmental organization (6)
- Ethics review committee not linked to another institution / organization (7)
- OTHER (please specify in the text box below): (8) ________________________________________________

| Page Break |  |
| --- | --- |

I How long have you been a member of the ethics review committee?

- 1 year or less (1)
- 2 - 5 years (2)
- 6 - 10 years (3)
- 11 - 15 years (4)
- 16 - 20 years (5)
- 21 or more years (6)

End of Block: EXPERIENCE WITH ETHICS REVIEWS

Start of Block: EXISTING POLICIES AND GUIDELINES

1 Prior to the COVID-19 pandemic, did your ethics review committee have any policies, procedures, or guidelines concerning how the committee should operate during a public health emergency, like a pandemic?

- Yes (1)
- No (2)
- Unsure (3)

Skip To: 2 If Prior to the COVID-19 pandemic, did your ethics review committee have any policies, procedures, o... = No

Skip To: 2 If Prior to the COVID-19 pandemic, did your ethics review committee have any policies, procedures, o... = Unsure

| Page Break |  |
| --- | --- |

1a If it is possible to share, and without breaking your duty of confidentiality, please attach the relevant policy, procedure, or guideline. Otherwise, please describe the corresponding document or provide a link to it in the text box below.

1b Please briefly describe the corresponding document or provide a link to it here, taking care to avoid sharing confidential information:

________________________________________________________________

________________________________________________________________

________________________________________________________________

________________________________________________________________

________________________________________________________________

| Page Break |  |
| --- | --- |

2 Since the beginning of the pandemic, did your ethics review committee use any pre-existing, **external** guidelines (i.e., not developed by your ethics review committee) to govern its operation during public health emergencies, like a pandemic?

- Yes (1)
- No (2)
- Unsure (3)

Skip To: End of Block If Since the beginning of the pandemic, did your ethics review committee use any pre-existing, exter... = No

Skip To: End of Block If Since the beginning of the pandemic, did your ethics review committee use any pre-existing, exter... = Unsure

| Page Break |  |
| --- | --- |

2a If possible, and without breaking your duty of confidentiality, please attach the relevant policy, procedure, or guideline. Otherwise, please describe the corresponding document or provide a link to it in the text box below.

2b Please briefly describe the corresponding document or provide a link to it here, taking care to avoid sharing confidential information:

________________________________________________________________

________________________________________________________________

________________________________________________________________

________________________________________________________________

________________________________________________________________

End of Block: EXISTING POLICIES AND GUIDELINES

Start of Block: MODIFIED AND NEW PROCEDURES

| Page Break |  |
| --- | --- |

3 Did your ethics review committee modify **EXISTING** procedures or policies to adapt to the COVID-19 pandemic? *(For the purposes of this study, by “modifying existing procedures or policies” we mean making changes to procedures or policies already in use by your ethics review committee before March 2020 as a means of adapting to the new circumstances brought about by the COVID-19 pandemic.)*

- Yes (1)
- No (2)
- Unsure (3)

Skip To: 4 If Did your ethics review committee modify EXISTING procedures or policies to adapt to the COVID-19... = No

Skip To: 4 If Did your ethics review committee modify EXISTING procedures or policies to adapt to the COVID-19... = Unsure

| Page Break |  |
| --- | --- |

3a Please select all procedures or policies to which modifications were made:

- Protocol review (including modifying the criteria to determine which projects required full review and which could simply undergo expedited review) (1)
- Procedures for protocol approval (2)
- Pre-screening of research protocols (3)
- Availability of generic research protocols in advance of the pandemic so that they could be rapidly modified for specific settings (4)
- Reduction of documentation requirements (5)
- Meeting logistics (including teleconferencing capabilities and virtual meetings) (6)
- Meeting frequency (7)
- Security for ethics review committee members (8)
- Data management and storage (9)
- Training of ethics review committee members in new or modified procedures for research protocol review (10)
- Hiring additional administrative staff to manage increased workload (11)
- Establishing sub-committees focused exclusively on COVID-19-related projects (12)
- OTHER (please specify in the text box below): (13) ________________________________________________

| Page Break |  |
| --- | --- |

3b Has your ethics review committee conducted an evaluation of the success or failure of any **modified procedures or policies** implemented during the pandemic?

- Yes (1)
- No (2)
- Unsure (3)

| Page Break |  |
| --- | --- |

4 Did your ethics review committee design and implement **NEW** procedures or other innovations to adapt to the COVID-19 pandemic? *(For the purposes of this study, “new procedures or other innovations” means the design and implementation of novel procedures or policies that did not exist before March 2020 and thus could not have been used by your ethics review committee before this date.)*

- Yes (1)
- No (2)
- Unsure (3)

Skip To: 5 If Did your ethics review committee design and implement NEW procedures or other innovations to adap... = No

Skip To: 5 If Did your ethics review committee design and implement NEW procedures or other innovations to adap... = Unsure

| Page Break |  |
| --- | --- |

4a Please select all new procedures that apply:

- Protocol review (including modifying the criteria to determine which projects required full review and which could simply undergo expedited review) (1)
- Procedures for protocol approval (2)
- Pre-screening of research protocols (3)
- Availability of generic research protocols in advance of the pandemic so that they could be rapidly modified for specific settings (4)
- Reduction of documentation requirements (5)
- Meeting logistics (including teleconferencing capabilities and virtual meetings) (6)
- Meeting frequency (7)
- Security for ethics review committee members (8)
- Data management and storage (9)
- Training of ethics review committee members in new or modified procedures for research protocol review (10)
- Hiring additional administrative staff to manage increased workload (11)
- Establishing sub-committees focused exclusively on COVID-19-related projects (12)
- OTHER (please specify in the text box below): (13) ________________________________________________

| Page Break |  |
| --- | --- |

4b Has your ethics review committee conducted an evaluation of the success or failure of any **new procedures or other innovations** designed and implemented during the pandemic?

- Yes (1)
- No (2)
- Unsure (3)

| Page Break |  |
| --- | --- |

5 Do you believe that some of the modifications or innovations to operating procedures implemented by your ethics review committee during the pandemic should be put into effect permanently to improve everyday functioning?

- Yes (1)
- No (2)
- Unsure (3)
- Not applicable (4)

Skip To: 7 If Do you believe that some of the modifications or innovations to operating procedures implemented... = No

Skip To: 7 If Do you believe that some of the modifications or innovations to operating procedures implemented... = Unsure

Skip To: 7 If Do you believe that some of the modifications or innovations to operating procedures implemented... = Not applicable

| Page Break |  |
| --- | --- |

5a Please select all modifications or innovations to operating procedures implemented by your ethics review committee during the pandemic that, in your view, should be put into effect permanently:

- Protocol review (including modifying the criteria to determine which projects required full review and which could simply undergo expedited review) (1)
- Procedures for protocol approval (2)
- Pre-screening of research protocols (3)
- Availability of generic research protocols in advance of the pandemic so that they could be rapidly modified for specific settings (4)
- Reduction of documentation requirements (5)
- Meeting logistics (including teleconferencing capabilities and virtual meetings) (6)
- Meeting frequency (7)
- Security for ethics review committee members (8)
- Data management and storage (9)
- Training of ethics review committee members in new or modified procedures for research protocol review (10)
- Hiring additional administrative staff to manage increased workload (11)
- Establishing sub-committees focused exclusively on COVID-19-related projects (12)
- OTHER (please specify in the text box below): (13) ________________________________________________

| Page Break |  |
| --- | --- |

7 Does your ethics review committee have support (financial or otherwise) to permanently implement any modifications or innovations to operating procedures established during the pandemic?

- Yes (1)
- No (2)
- Unsure (3)

End of Block: MODIFIED AND NEW PROCEDURES

Start of Block: NATIONAL, INTERNATIONAL, AND MULTICENTRE COLLABORATION

8 Did the country in which your ethics review committee resides implement any form of centralized ethics review of research protocols for multicentre studies related to COVID-19?

- Yes (1)
- No (2)
- Unsure (3)

| Page Break |  |
| --- | --- |

9 Did your ethics review committee develop and implement strategies to harmonize multiple review processes within and across countries, especially between those of different income levels?

- Yes (1)
- No (2)
- Unsure (3)

| Page Break |  |
| --- | --- |

10 Did your ethics review committee collaborate with other ethics committees nationally or internationally to standardize emergency operations and procedures during the pandemic?

- Yes (1)
- No (2)
- Unsure (3)

| Page Break |  |
| --- | --- |

11 Did your ethics review committee consider the formation of Joint Scientific Advisory Committees, Data Safety Review Committees, Data Access Committees, or a Joint Ethics Review Committee with representatives of ethics committees of all institutions and countries involved in COVID-19-related research?

- Yes (1)
- No (2)
- Unsure (3)

| Page Break |  |
| --- | --- |

12 Did your ethics review committee rely on established procedures to recognize or validate research protocol reviews conducted by other ethics committees within your country or in other countries?

- Yes (1)
- No (2)
- Unsure (3)

| Page Break |  |
| --- | --- |

13 Did your ethics review committee collaborate with scientific committees that pre-reviewed or prioritized pandemic-related research protocols?

- Yes (1)
- No (2)
- Unsure (3)

| Page Break |  |
| --- | --- |

14 Did your ethics review committee receive pressure in any way or form by governments, organizations, sponsors, corporations, researchers, or any other entity, to approve or reject specific research protocols?

- Yes (please describe in the text box below, if possible, taking care to avoid breaking your duty of confidentiality) (1) ________________________________________________
- No (2)
- Unsure (3)

| Page Break |  |
| --- | --- |

End of Block: NATIONAL, INTERNATIONAL, AND MULTICENTRE COLLABORATION

Start of Block: CHALLENGES RELATED TO ETHICS COMMITTEE MEMBERS

15 Was it difficult for your ethics review committee to ensure a quorum was met during meetings held during the COVID-19 pandemic?

- No, quorum was always met (1)
- Infrequently (2)
- Frequently (3)
- Unsure (4)

| Page Break |  |
| --- | --- |
|  |  |

16 Did your ethics review committee take measures to ensure continuity of adequate review of research protocols in case existing members became unavailable due to the pandemic?

- Yes (1)
- No (2)
- Unsure (3)

| Page Break |  |
| --- | --- |

17 Did your ethics review committee add new members to accelerate protocol review during the COVID-19 pandemic?

- Yes (1)
- No (2)
- Unsure (3)

| Page Break |  |
| --- | --- |

18 Did your ethics review committee add new members with specific expertise to address novel areas of research or provide enhanced scrutiny of research protocols during the COVID-19 pandemic?

- Yes (1)
- No (2)
- Unsure (3)

| Page Break |  |
| --- | --- |

19 Did your ethics review committee consult expert non-members to address novel areas of research or provide enhanced scrutiny of research protocols during the COVID-19 pandemic?

- Yes (1)
- No (2)
- Unsure (3)

End of Block: CHALLENGES RELATED TO ETHICS COMMITTEE MEMBERS

Start of Block: COMPARISON BETWEEN OPERATIONS BEFORE AND DURING THE PANDEMIC

| Page Break |  |
| --- | --- |

20 Compared to the volume of research protocols typically reviewed by the ethics committee before the pandemic (e.g., volume of protocols reviewed per month before March 2020), please approximate the extent of change in the volume of protocols reviewed during the pandemic (including both COVID-19 and non-COVID-19 research protocols):

|  | **Decreased 100%** (1) | **Decreased 51% - 99%** (2) | **Decreased 1% - 50%** (3) | **Remained about the same** (4) | **Increased 1% - 50%** (5) | **Increased 51% - 99%** (6) | **Increased more than 100%** (7) |
| --- | --- | --- | --- | --- | --- | --- | --- |
| **Delegated or expedited review** (1) |  |  |  |  |  |  |  |
| **Full review** (2) |  |  |  |  |  |  |  |

| Page Break |  |
| --- | --- |

21 On average, **prior to the pandemic**, how long did it take for research protocols to be approved, from the time of initial submission to full approval?

|  | **<2 weeks** (1) | **3-5 weeks** (2) | **6-8 weeks** (3) | **9-11 weeks** (4) | **>12 weeks** (5) | **Unsure / Time not tracked** (6) |
| --- | --- | --- | --- | --- | --- | --- |
| **Delegated or expedited review** (1) |  |  |  |  |  |  |
| **Full review** (2) |  |  |  |  |  |  |

| Page Break |  |
| --- | --- |

22 On average, how long did it take for **COVID-19-related research protocols** to be approved during the pandemic, from the time of initial submission to full approval?

|  | **<2 weeks** (1) | **3-5 weeks** (2) | **6-8 weeks** (3) | **9-11 weeks** (4) | **>12 weeks** (5) | **Unsure / Time not tracked** (6) |
| --- | --- | --- | --- | --- | --- | --- |
| **Delegated or expedited review** (1) |  |  |  |  |  |  |
| **Full review** (2) |  |  |  |  |  |  |

| Page Break |  |
| --- | --- |

23 On average, how long did it take for **non-COVID-19-related research protocols** to be approved during the pandemic, from the time of initial submission to full approval?

|  | **<2 weeks** (1) | **3-5 weeks** (2) | **6-8 weeks** (3) | **9-11 weeks** (4) | **>12 weeks** (5) | **Unsure / Time not tracked** (6) |
| --- | --- | --- | --- | --- | --- | --- |
| **Delegated or expedited review** (1) |  |  |  |  |  |  |
| **Full review** (2) |  |  |  |  |  |  |

| Page Break |  |
| --- | --- |

24 On average, how long did it take your ethics review committee to complete the review of research protocols during the pandemic? That is, how long, on average, were protocols in the hands of ethics review committee members for review (not counting the time it took researchers to respond to the committee’s comments, suggestions, and observations)?

|  | **Less than 7 days** (1) | **Between 8-14 days** (2) | **2 to 4 weeks** (3) | **4 to 8 weeks** (4) | **8 to 12 weeks** (5) | **More than 12 weeks** (6) | **Unsure / Time not tracked** (7) |
| --- | --- | --- | --- | --- | --- | --- | --- |
| **Delegated or expedited review** (1) |  |  |  |  |  |  |  |
| **Full review** (2) |  |  |  |  |  |  |  |

| Page Break |  |
| --- | --- |

25 If available, please indicate the **number of COVID-19-related research protocols** that your ethics review committee has reviewed, according to the type of study:

|  | **0** (1) | **1 - 10** (2) | **11 - 20** (3) | **21 - 30** (4) | **> 30** (5) | **Info not available** (6) |
| --- | --- | --- | --- | --- | --- | --- |
| **Diagnostics** (1) |  |  |  |  |  |  |
| **Therapeutics** (2) |  |  |  |  |  |  |
| **Vaccines** (3) |  |  |  |  |  |  |
| **Pharmacovigilance** (4) |  |  |  |  |  |  |
| **Other (e.g., behavioural or social sciences):** (5) |  |  |  |  |  |  |

| Page Break |  |
| --- | --- |

26 Did your ethics review committee prioritize the review of COVID-19-related research over non-COVID-19-related research during the pandemic?

- Yes (1)
- No (2)
- Unsure (3)

| Page Break |  |
| --- | --- |

27 Did your ethics review committee prioritize the review of some types of COVID-19-related research over others during the pandemic?

- Yes (please explain in the text box below, taking care to avoid breaking your duty of confidentiality): (1) ________________________________________________
- No (2)
- Unsure (3)

End of Block: COMPARISON BETWEEN OPERATIONS BEFORE AND DURING THE PANDEMIC

Start of Block: SUPPORT RECEIVED FOR OPERATIONS DURING THE PANDEMIC

28 Did your ethics review committee receive additional support for its operations during the pandemic?

- Yes (1)
- No (2)

Skip To: 29 If Did your ethics review committee receive additional support for its operations during the pandemic? = No

28a Please select all the types of additional support your ethics review committee received:

- Financial (1)
- Human resources (2)
- Administrative resources (3)
- OTHER (please specify in the text box below): (4) ________________________________________________

| Page Break |  |
| --- | --- |

28b Please select all the areas that received additional support during the pandemic:

- Ethics committee support staff (1)
- Ethics committee reviewers (2)
- External experts (3)
- Teleconferencing and virtual meeting capabilities (4)
- Information technology (IT) support (5)
- Training of ethics committee members (6)
- Security for ethics committee members (7)
- Data management and storage (8)
- Other administrative costs (9)
- OTHER (please specify in the text box below): (10) ________________________________________________

| Page Break |  |
| --- | --- |

29 **Prior to the pandemic**, did financial planning include provisions for emergency funds or other supports for the operation of your ethics review committee during a public health emergency?

- Yes (1)
- No (2)
- Unsure (3)

End of Block: SUPPORT RECEIVED FOR OPERATIONS DURING THE PANDEMIC

Start of Block: CONCLUDING QUESTIONS

30 Is there anything not previously covered in this survey, including additional challenges faced by your ethics review committee, that you would like to communicate to us regarding your experiences conducting research ethics reviews during the COVID-19 pandemic (without breaking your duty of confidentiality)?

________________________________________________________________

________________________________________________________________

________________________________________________________________

________________________________________________________________

________________________________________________________________

| Page Break |  |
| --- | --- |

End of Block: CONCLUDING QUESTIONS

| **THANK YOU MESSAGE FOR RESPONDENTS WHO COMPLETED THE SURVEY:**  **Thank you very much for completing our survey! Your answers have been recorded.**  **We are certain that your participation will contribute to understanding the role and functioning of ethics review committees during the COVID-19 pandemic. Your contribution will also help identify which successful strategies designed and implemented by these committees globally can guide approaches to future public health emergencies.** |
| --- |
