# Supplementary material for: Research ethics review during the COVID-19 pandemic: An international study
Source: PLoS One. 2024 Apr 16;19(4):e0292512. doi: 10.1371/journal.pone.0292512 (PMC11020390; doi:10.1371/journal.pone.0292512)
Supplement: S2 Appendix — (DOCX) [file pone.0292512.s002.docx]

**Research ethics review during the COVID-19 pandemic: An international study**

**Supporting Information**

**S2 Appendix. Supplementary Tables**

Table S 1. World Health Organization region for which ethics review was provided

| **World Health Organization region** | **HICs** | | **LMICs** | | **TOTAL** | |  |
| --- | --- | --- | --- | --- | --- | --- | --- |
|  | **n** | **%** | **n** | **%** | **n** | **%** |  |
| **Europe** | 77 | 53.0 | 7 | 8.0 | 84 | 36.1 |  |
| **Americas** | 14 | 10.0 | 28 | 32.2 | 42 | 18.0 |  |
| **Africa** | 4 | 3.0 | 17 | 19.5 | 21 | 9.0 |  |
| **South-East Asia** | 8 | 5.0 | 10 | 11.5 | 18 | 7.7 |  |
| **Eastern Mediterranean** | 0 | 0.0 | 8 | 9.2 | 8 | 3.4 |  |
| **Western Pacific** | 3 | 2.0 | 0 | 0.0 | 3 | 1.3 |  |
| **Only reviewed research protocols from own country** | 40 | 27.0 | 17 | 19.5 | 57 | 24.5 |  |
| **TOTAL** | 146 | 100.0 | 87 | 100.0 | 233 | 100.0 |  |
| ***Note: For this question, respondents could select more than one category. Therefore, "totals" refer to number of selections, not of respondents*** | | | | | | | |

Table S 2. Length of experience of participants as ethics review committee members

| **Length of experience in RECs** | **HICs** | | **LMICs** | | **TOTAL** | |
| --- | --- | --- | --- | --- | --- | --- |
|  | **n** | **%** | **n** | **%** | **n** | **%** |
| **1 year or less** | 3 | 2.4 | 3 | 4.3 | 6 | 3.1 |
| **2 - 5 years** | 41 | 32.8 | 19 | 27.1 | 60 | 30.8 |
| **6 - 10 years** | 40 | 32.0 | 30 | 42.9 | 70 | 35.9 |
| **11 - 15 years** | 17 | 13.6 | 11 | 15.7 | 28 | 14.4 |
| **16 - 20 years** | 9 | 7.2 | 4 | 5.7 | 13 | 6.7 |
| **21 or more years** | 15 | 12.0 | 3 | 4.3 | 18 | 9.2 |
| **TOTAL** | 125 | 100.0 | 70 | 100.0 | 195 | 100.0 |

Table S 3. Number of ethics review committees in which participants were involved during the COVID-19 pandemic

|  | **HICs** | | **LMICs** | | **TOTAL** | |
| --- | --- | --- | --- | --- | --- | --- |
|  | **n** | **%** | **n** | **%** | **n** | **%** |
| **One** | 65 | 50.4 | 39 | 54.9 | 104 | 52.0 |
| **Two** | 17 | 13.2 | 16 | 22.5 | 33 | 16.5 |
| **Three or more** | 47 | 36.4 | 16 | 23 | 63 | 31.5 |
| **TOTAL** | 129 | 100.0 | 71 | 100.0 | 200 | 100.0 |

Table S 4. Simultaneous involvement with multiple ethics review committees

|  | | | | | | |
| --- | --- | --- | --- | --- | --- | --- |
|  | **HICs** | | **LMICs** | | **TOTAL** | |
|  | **n** | **%** | **n** | **%** | **n** | **%** |
| **Yes** | 32 | 50.0 | 18 | 56.3 | 50 | 52.1 |
| **No** | 32 | 50.0 | 14 | 43.8 | 46 | 47.9 |
| **TOTAL** | 64 | 100.0 | 32 | 100.0 | 96 | 100.0 |

**Table S 5. Presence of additional support for the operation of ethics review committee during the COVID-19 pandemic**

|  | **HICs** | | **LMICs** | | **TOTAL** | |
| --- | --- | --- | --- | --- | --- | --- |
|  | **n** | **%** | **n** | **%** | **n** | **%** |
| **Yes** | 27 | 26.0 | 8 | 13.8 | 35 | 21.6 |
| **No** | 77 | 74.0 | 50 | 86.2 | 127 | 78.4 |
| **TOTAL** | 104 | 100.0 | 58 | 100.0 | 162 | 100.0 |

**Table S 6. Additional support of ethics review committee, by type**

|  | **HICs** | | **LMICs** | | **TOTAL** | |
| --- | --- | --- | --- | --- | --- | --- |
|  | **n** | **%** | **n** | **%** | **n** | **%** |
| **Administrative resources** | 22 | 55.0 | 6 | 35.3 | 28 | 49.1 |
| **Human resources** | 8 | 20.0 | 4 | 23.5 | 12 | 21.1 |
| **Financial** | 5 | 12.5 | 4 | 23.5 | 9 | 15.8 |
| **Other** | 5 | 12.5 | 3 | 17.6 | 8 | 14.0 |
| **TOTAL** | 40 | 100.0 | 17 | 100.0 | 57 | 100.0 |

***Note: For this question, respondents could select more than one category. Therefore, "totals" refer to number of selections, not of respondents***

**Table S 7. Pre-pandemic financial planning with provisions for support of ethics review committees during a public health emergency**

|  | **HICs** | | **LMICs** | | **TOTAL** | |
| --- | --- | --- | --- | --- | --- | --- |
|  | **n** | **%** | **n** | **%** | **n** | **%** |
| **Yes** | 4 | 3.8 | 2 | 3.4 | 6 | 3.7 |
| **No** | 46 | 43.8 | 53 | 91.4 | 99 | 60.7 |
| **Unsure** | 55 | 52.4 | 3 | 5.2 | 58 | 35.6 |
| **TOTAL** | 105 | 100.0 | 58 | 100.0 | 163 | 100.0 |

**Table S 8. Modification of existing procedures or policies**

|  | **HICs** | | **LMICs** | | **TOTAL** | |
| --- | --- | --- | --- | --- | --- | --- |
|  | **n** | **%** | **n** | **%** | **n** | **%** |
| **Yes** | 91 | 75.8 | 49 | 77.8 | 140 | 76.5 |
| **No** | 13 | 10.8 | 11 | 17.5 | 24 | 13.1 |
| **Unsure** | 16 | 13.3 | 3 | 4.8 | 19 | 10.4 |
| **TOTAL** | 120 | 100.0 | 63 | 100.0 | 183 | 100.0 |

**Table S 9. Modified procedures and policies**

|  | **HICs** | | **LMICs** | | **TOTAL** | |
| --- | --- | --- | --- | --- | --- | --- |
|  | **n** | **%** | **n** | **%** | **n** | **%** |
| **Meeting logistics** | 71 | 26.9 | 45 | 18.8 | 116 | 23.1 |
| **Meeting frequency** | 35 | 13.3 | 29 | 12.1 | 64 | 12.7 |
| **Protocol review** | 32 | 12.1 | 28 | 11.7 | 60 | 11.9 |
| **Procedures for protocol approval** | 26 | 9.8 | 27 | 11.3 | 53 | 10.5 |
| **Training of ethics review committee members in new or modified procedures** | 26 | 9.8 | 22 | 9.2 | 48 | 9.5 |
| **Establishing sub-committees focused exclusively on COVID-19-related projects** | 26 | 9.8 | 14 | 5.9 | 40 | 8.0 |
| **Pre-screening of research protocols** | 12 | 4.5 | 18 | 7.5 | 30 | 6.0 |
| **Data management and storage** | 8 | 3.0 | 15 | 6.3 | 23 | 4.6 |
| **Reduction of documentation requirements** | 7 | 2.7 | 12 | 5.0 | 19 | 3.8 |
| **Security for ethics review committee members** | 4 | 1.5 | 10 | 4.2 | 14 | 2.8 |
| **Availability of generic research protocols in advance of the pandemic** | 3 | 1.1 | 10 | 4.2 | 13 | 2.6 |
| **Hiring additional administrative staff to manage increased workload** | 1 | 0.4 | 3 | 1.3 | 4 | 0.8 |
| **Other** | 13 | 4.9 | 6 | 2.5 | 19 | 3.8 |
| **TOTAL** | 264 | 100.0 | 239 | 100.0 | 503 | 100.0 |

***Note: For this question, respondents could select more than one category. Therefore, "totals" refer to number of selections, not of respondents***

**Table S 10. Design and implementation of new procedures and policies**

|  | **HICS** | | **LMICS** | | **TOTAL** | |
| --- | --- | --- | --- | --- | --- | --- |
| **CATEGORY** | **n** | **%** | **n** | **%** | **n** | **%** |
| **Yes** | 68 | 59.6 | 37 | 60.7 | 105 | 60.0 |
| **No** | 27 | 23.7 | 17 | 27.9 | 44 | 25.1 |
| **Unsure** | 19 | 16.7 | 7 | 11.5 | 26 | 14.9 |
| **TOTAL** | 114 | 100.0 | 61 | 100.0 | 175 | 100.0 |

**Table S 11. New procedures and policies**

|  | **HICs** | | **LMICs** | | **TOTAL** | |
| --- | --- | --- | --- | --- | --- | --- |
|  | **n** | **%** | **n** | **%** | **n** | **%** |
| **Meeting logistics** | 52 | 27.2 | 32 | 18.7 | 84 | 23.2 |
| **Meeting frequency** | 24 | 12.6 | 26 | 15.2 | 50 | 13.8 |
| **Procedures for protocol approval** | 21 | 11.0 | 25 | 14.6 | 46 | 12.7 |
| **Protocol review** | 24 | 12.6 | 20 | 11.7 | 44 | 12.2 |
| **Pre-screening of research protocols** | 16 | 8.4 | 14 | 8.2 | 30 | 8.3 |
| **Training of ethics review committee members in new or modified procedures** | 14 | 7.3 | 12 | 7.0 | 26 | 7.2 |
| **Establishing sub-committees focused exclusively on COVID-19-related projects** | 14 | 7.3 | 4 | 2.3 | 18 | 5.0 |
| **Reduction of documentation requirements** | 5 | 2.6 | 10 | 5.8 | 15 | 4.1 |
| **Data management and storage** | 4 | 2.1 | 10 | 5.8 | 14 | 3.9 |
| **Security for ethics review committee members** | 5 | 2.6 | 9 | 5.3 | 14 | 3.9 |
| **Availability of generic research protocols in advance of the pandemic** | 4 | 2.1 | 4 | 2.3 | 8 | 2.2 |
| **Hiring additional administrative staff to manage increased workload** | 1 | 0.5 | 2 | 1.2 | 3 | 0.8 |
| **Other** | 7 | 3.7 | 3 | 1.8 | 10 | 2.8 |
| **TOTAL** | 191 | 100.0 | 171 | 100.0 | 362 | 100.0 |

***Note: For this question, respondents could select more than one category. Therefore, "totals" refer to number of selections, not of respondents***

**Table S 12. Opinions on whether to permanently put into effect modifications or innovations to operating procedures implemented during the COVID-19 pandemic**

|  | **HICs** | | **LMICs** | | **TOTAL** | |
| --- | --- | --- | --- | --- | --- | --- |
|  | **n** | **%** | **n** | **%** | **n** | **%** |
| **Yes** | 71 | 72.4 | 39 | 81.3 | 110 | 75.3 |
| **No** | 10 | 10.2 | 5 | 10.4 | 15 | 10.3 |
| **Unsure** | 17 | 17.3 | 4 | 8.3 | 21 | 14.4 |
| **TOTAL** | 98 | 100.0 | 48 | 100.0 | 146 | 100.0 |

Table S 13. Modifications or innovations to operating procedures implemented during the COVID-19 pandemic that should be permanently put into effect

|  | **HICs** | | **LMICs** | | **TOTAL** | |
| --- | --- | --- | --- | --- | --- | --- |
|  | **n** | **%** | **n** | **%** | **n** | **%** |
| **Meeting logistics** | 53 | 32.1 | 36 | 22.8 | 89 | 27.6 |
| **Protocol review** | 21 | 12.7 | 16 | 10.1 | 37 | 11.5 |
| **Training of ethics review committee members in new or modified procedures** | 18 | 10.9 | 14 | 8.9 | 32 | 9.9 |
| **Procedures for protocol approval** | 9 | 5.5 | 19 | 12.0 | 28 | 8.7 |
| **Meeting frequency** | 10 | 6.1 | 17 | 10.8 | 27 | 8.4 |
| **Pre-screening of research protocols** | 11 | 6.7 | 14 | 8.9 | 25 | 7.7 |
| **Data management and storage** | 7 | 4.2 | 10 | 6.3 | 17 | 5.3 |
| **Reduction of documentation requirements** | 7 | 4.2 | 8 | 5.1 | 15 | 4.6 |
| **Hiring additional administrative staff to manage increased workload** | 5 | 3.0 | 8 | 5.1 | 13 | 4.0 |
| **Availability of generic research protocols in advance of the pandemic** | 7 | 4.2 | 5 | 3.2 | 12 | 3.7 |
| **Establishing sub-committees focused exclusively on COVID-19-related projects** | 8 | 4.8 | 4 | 2.5 | 12 | 3.7 |
| **Security for ethics review committee members** | 7 | 4.2 | 4 | 2.5 | 11 | 3.4 |
| **Other** | 2 | 1.2 | 3 | 1.9 | 5 | 1.5 |
| **TOTAL** | 165 | 100.0 | 158 | 100.0 | 323 | 100.0 |

***Note: For this question, respondents could select more than one category. Therefore, "totals" refer to number of selections, not of respondents***

**Table S 14. Presence of support to permanently implement modifications or innovations established during the COVID-19 pandemic**

|  | **HICs** | | **LMICs** | | **TOTAL** | |
| --- | --- | --- | --- | --- | --- | --- |
|  | **n** | **%** | **n** | **%** | **n** | **%** |
| **Yes** | 44 | 40.0 | 20 | 33.3 | 64 | 37.6 |
| **No** | 22 | 20.0 | 30 | 50.0 | 52 | 30.6 |
| **Unsure** | 44 | 40.0 | 10 | 16.7 | 54 | 31.8 |
| **TOTAL** | 110 | 100.0 | 60 | 100.0 | 170 | 100.0 |

**Table S 15. Presence of internal policies, procedures, or guidelines**

|  | **HICs** | | **LMICs** | | **TOTAL** | |
| --- | --- | --- | --- | --- | --- | --- |
|  | **n** | **%** | **n** | **%** | **n** | **%** |
| **Yes** | 17 | 13.6 | 13 | 18.6 | 30 | 15.4 |
| **No** | 58 | 46.4 | 52 | 74.3 | 110 | 56.4 |
| **Unsure** | 50 | 40.0 | 5 | 7.1 | 55 | 28.2 |
| **TOTAL** | 125 | 100.0 | 70 | 100.0 | 195 | 100.0 |

Table S 16. Presence of external policies, procedures, or guidelines

|  | **HICs** | | **LMICs** | | **TOTAL** | |
| --- | --- | --- | --- | --- | --- | --- |
|  | **n** | **%** | **n** | **%** | **n** | **%** |
| **Yes** | 32 | 26.4 | 32 | 45.7 | 64 | 33.5 |
| **No** | 43 | 35.5 | 31 | 44.3 | 74 | 38.7 |
| **Unsure** | 46 | 38.0 | 7 | 10.0 | 53 | 27.7 |
| **TOTAL** | 121 | 100.0 | 70 | 100.0 | 191 | 100.0 |

**Table S 17. Extent of change in the volume of protocols reviewed during the pandemic**

|  | **Delegated / expedited review** | | | | **Full review** | | | |
| --- | --- | --- | --- | --- | --- | --- | --- | --- |
|  | **HICs** | | **LMICs** | | **HICs** | | **LMICs** | |
|  | **n** | **%** | **n** | **%** | **n** | **%** | **n** | **%** |
| **Decreased 100%** | 1 | 1.0 | 0 | 0.0 | 1 | 1.0 | 0 | 0.0 |
| **Decreased 51 – 99%** | 0 | 0.0 | 2 | 3.6 | 0 | 0.0 | 6 | 10.7 |
| **Decreased 1% – 50%** | 5 | 5.2 | 3 | 5.4 | 10 | 10.3 | 10 | 17.9 |
| **Remained about the same** | 38 | 39.2 | 15 | 26.8 | 42 | 43.3 | 12 | 21.4 |
| **Increased 1% – 50%** | 38 | 39.2 | 20 | 35.7 | 32 | 33.0 | 19 | 33.9 |
| **Increased 51% - 99%** | 13 | 13.4 | 12 | 21.4 | 12 | 12.4 | 5 | 8.9 |
| **Increased more than 100%** | 2 | 2.1 | 4 | 7.1 | 0 | 0.0 | 4 | 7.1 |
| **TOTAL** | 97 | 100.0 | 56 | 100.0 | 97 | 100.0 | 56 | 100.0 |

**Table S 18. Time it took before the COVID-19 pandemic for research protocols to be approved, from the time of initial submission to full approval**

|  | **Delegated / expedited review** | | | | **Full review** | | | |
| --- | --- | --- | --- | --- | --- | --- | --- | --- |
|  | **HICs** | | **LMICs** | | **HICs** | | **LMICs** | |
|  | **n** | **%** | **n** | **%** | **n** | **%** | **n** | **%** |
| **≤ 2 weeks** | 17 | 17.3 | 16 | 28.6 | 4 | 4.0 | 2 | 3.4 |
| **3-5 weeks** | 39 | 39.8 | 22 | 39.3 | 29 | 29.0 | 22 | 37.9 |
| **6-8 weeks** | 11 | 11.2 | 8 | 14.3 | 30 | 30.0 | 18 | 31.0 |
| **9-11 weeks** | 1 | 1.0 | 4 | 7.1 | 9 | 9.0 | 5 | 8.6 |
| **≥ 12 weeks** | 2 | 2.0 | 1 | 1.8 | 8 | 8.0 | 8 | 13.8 |
| **Unsure / Time not tracked** | 28 | 28.6 | 5 | 8.9 | 20 | 20.0 | 3 | 5.2 |
| **TOTAL** | 98 | 100.0 | 56 | 100.0 | 100 | 100.0 | 58 | 100.0 |

**Table S 19. Length of time that ethics review committee members took to complete review of research protocols during the COVID-19 pandemic**

|  | **Delegated / expedited review** | | | | **Full review** | | | |
| --- | --- | --- | --- | --- | --- | --- | --- | --- |
|  | **HICs** | | **LMICs** | | **HICs** | | **LMICs** | |
|  | **n** | **%** | **n** | **%** | **n** | **%** | **n** | **%** |
| **≤ 7 days** | 43 | 43.4 | 17 | 30.4 | 21 | 20.8 | 9 | 15.3 |
| **8-14 days** | 37 | 37.4 | 13 | 23.2 | 49 | 48.5 | 14 | 23.7 |
| **2 to 4 weeks** | 10 | 10.1 | 12 | 21.4 | 17 | 16.8 | 13 | 22.0 |
| **4 to 8 weeks** | 1 | 1.0 | 9 | 16.1 | 5 | 5.0 | 15 | 25.4 |
| **8 to 12 weeks** | 1 | 1.0 | 1 | 1.8 | 2 | 2.0 | 2 | 3.4 |
| **≥ 12 weeks** | 0 | 0.0 | 1 | 1.8 | 0 | 0.0 | 2 | 3.4 |
| **Unsure / Time not tracked** | 7 | 7.1 | 3 | 5.4 | 7 | 6.9 | 4 | 6.8 |
| **TOTAL** | 99 | 100.0 | 56 | 100.0 | 101 | 100.0 | 59 | 100.0 |

**Table S 20. Total time to approval of COVID-19-related research protocols**

|  | **Delegated / expedited review** | | | | **Full review** | | | |
| --- | --- | --- | --- | --- | --- | --- | --- | --- |
|  | **HICs** | | **LMICs** | | **HICs** | | **LMICs** | |
|  | **n** | **%** | **n** | **%** | **n** | **%** | **n** | **%** |
| **≤ 2 weeks** | 44 | 45.4 | 28 | 50.9 | 28 | 27.7 | 18 | 31.0 |
| **3-5 weeks** | 24 | 24.7 | 12 | 21.8 | 32 | 31.7 | 17 | 29.3 |
| **6-8 weeks** | 6 | 6.2 | 10 | 18.2 | 16 | 15.8 | 12 | 20.7 |
| **9-11 weeks** | 1 | 1.0 | 0 | 0.0 | 2 | 2.0 | 2 | 3.4 |
| **≥ 12 weeks** | 2 | 2.1 | 2 | 3.6 | 4 | 4.0 | 3 | 5.2 |
| **Unsure / Time not tracked** | 20 | 20.6 | 3 | 5.5 | 19 | 18.8 | 6 | 10.3 |
| **TOTAL** | 97 | 100.0 | 55 | 100.0 | 101 | 100.0 | 58 | 100.0 |

**Table S 21. Total time to approval for non-COVID-19-related research protocols**

|  | **Delegated / expedited review** | | | | **Full review** | | | |
| --- | --- | --- | --- | --- | --- | --- | --- | --- |
|  | **HICs** | | **LMICs** | | **HICs** | | **LMICs** | |
|  | **n** | **%** | **n** | **%** | **n** | **%** | **n** | **%** |
| **≤ 2 weeks** | 16 | 16.8 | 9 | 16.4 | 5 | 5.1 | 2 | 3.4 |
| **3-5 weeks** | 34 | 35.8 | 22 | 40.0 | 30 | 30.3 | 18 | 31.0 |
| **6-8 weeks** | 8 | 8.4 | 12 | 21.8 | 30 | 30.3 | 16 | 27.6 |
| **9-11 weeks** | 5 | 5.3 | 0 | 0.0 | 5 | 5.1 | 8 | 13.8 |
| **≥ 12 weeks** | 7 | 7.4 | 4 | 7.3 | 8 | 8.1 | 6 | 10.3 |
| **Unsure / Time not tracked** | 25 | 26.3 | 8 | 14.5 | 21 | 21.2 | 8 | 13.8 |
| **TOTAL** | 95 | 100.0 | 55 | 100.0 | 99 | 100.0 | 58 | 100.0 |

**Table S 22. Presence of external pressure on ethics review committees to approve or reject specific COVID-19 research protocols**

|  | **HICs** | | **LMICs** | | **TOTAL** | |
| --- | --- | --- | --- | --- | --- | --- |
|  | **n** | **%** | **n** | **%** | **n** | **%** |
| **Yes** | 15 | 13.9 | 20 | 33.9 | 35 | 21.0 |
| **No** | 72 | 66.7 | 35 | 59.3 | 107 | 64.1 |
| **Unsure** | 21 | 19.4 | 4 | 6.8 | 25 | 15.0 |
| **TOTAL** | 108 | 100.0 | 59 | 100.0 | 167 | 100.0 |

**Table S 23. Prioritization of COVID-19 related research over non-COVID-19-related protocols**

|  | **HICs** | | **LMICs** | | **TOTAL** | |
| --- | --- | --- | --- | --- | --- | --- |
|  | **n** | **%** | **n** | **%** | **n** | **%** |
| **Yes** | 80 | 76.2 | 38 | 64.4 | 118 | 72.0 |
| **No** | 16 | 15.2 | 16 | 27.1 | 32 | 19.5 |
| **Unsure** | 9 | 8.6 | 5 | 8.5 | 14 | 8.5 |
| **TOTAL** | 105 | 100.0 | 59 | 100.0 | 164 | 100.0 |

**Table S 24. Prioritization of some types of COVID-19-related research over others**

|  | **HICs** | | **LMICs** | | **TOTAL** | |
| --- | --- | --- | --- | --- | --- | --- |
|  | **n** | **%** | **n** | **%** | **n** | **%** |
| **Yes** | 32 | 31.1 | 25 | 42.4 | 57 | 35.2 |
| **No** | 49 | 47.6 | 23 | 39.0 | 72 | 44.4 |
| **Unsure** | 22 | 21.4 | 11 | 18.6 | 33 | 20.4 |
| **TOTAL** | 103 | 100.0 | 59 | 100.0 | 162 | 100.0 |

**Table S 25. Ensuring quorum**

|  | **HICs** | | **LMICs** | | **TOTAL** | |
| --- | --- | --- | --- | --- | --- | --- |
|  | **n** | **%** | **n** | **%** | **n** | **%** |
| Quorum was always met | 86 | 79.6 | 35 | 59.3 | 121 | 72.5 |
| Infrequently | 18 | 16.7 | 19 | 32.2 | 37 | 22.2 |
| Frequently | 3 | 2.8 | 4 | 6.8 | 7 | 4.2 |
| Unsure | 1 | 0.9 | 1 | 1.7 | 2 | 1.2 |
| **TOTAL** | 108 | 100.0 | 59 | 100.0 | 167 | 100.0 |

**Table S 26. Presence of measures to ensure continuity of adequate review of research protocols in case existing members became unavailable due to the pandemic**

|  | **HICs** | | **LMICs** | | **TOTAL** | |
| --- | --- | --- | --- | --- | --- | --- |
|  | **n** | **%** | **n** | **%** | **n** | **%** |
| **Yes** | 71 | 65.7 | 44 | 75.9 | 115 | 69.3 |
| **No** | 19 | 17.6 | 12 | 20.7 | 31 | 18.7 |
| **Unsure** | 18 | 16.7 | 2 | 3.4 | 20 | 12.0 |
| **TOTAL** | 108 | 100.0 | 58 | 100.0 | 166 | 100.0 |

**Table S 27. Addition of new members to accelerate protocol review during the COVID-19 pandemic**

|  | **HICs** | | **LMICs** | | **TOTAL** | |
| --- | --- | --- | --- | --- | --- | --- |
|  | **n** | **%** | **n** | **%** | **n** | **%** |
| **Yes** | 14 | 13.0 | 14 | 23.7 | 28 | 16.8 |
| **No** | 83 | 76.9 | 41 | 69.5 | 124 | 74.3 |
| **Unsure** | 11 | 10.2 | 4 | 6.8 | 15 | 9.0 |
| **TOTAL** | 108 | 100.0 | 59 | 100.0 | 167 | 100.0 |

**Table S 28. Addition of new members with specific expertise to address novel areas of research or provide enhanced scrutiny of research protocols during the COVID-19 pandemic**

|  | **HICs** | | **LMICs** | | **TOTAL** | |
| --- | --- | --- | --- | --- | --- | --- |
|  | **n** | **%** | **n** | **%** | **n** | **%** |
| **Yes** | 12 | 11.2 | 22 | 37.3 | 34 | 20.5 |
| **No** | 83 | 77.6 | 33 | 55.9 | 116 | 69.9 |
| **Unsure** | 12 | 11.2 | 4 | 6.8 | 16 | 9.6 |
| **TOTAL** | 107 | 100.0 | 59 | 100.0 | 166 | 100.0 |

**Table S 29. Consultation of expert non-members to address novel areas of research or provide enhanced scrutiny of research protocols during the COVID-19 pandemic**

|  | **HICs** | | **LMICs** | | **TOTAL** | |
| --- | --- | --- | --- | --- | --- | --- |
|  | **n** | **%** | **n** | **%** | **n** | **%** |
| **Yes** | 33 | 30.6 | 37 | 62.7 | 70 | 41.9 |
| **No** | 57 | 52.8 | 18 | 30.5 | 75 | 44.9 |
| **Unsure** | 18 | 16.7 | 4 | 6.8 | 22 | 13.2 |
| **TOTAL** | 108 | 100.0 | 59 | 100.0 | 167 | 100.0 |

Table S 30. National and international collaboration among ethics review committees to standardize emergency operations and procedures during the COVID-19 pandemic

|  | **HICs** | | **LMICs** | | **TOTAL** | |
| --- | --- | --- | --- | --- | --- | --- |
|  | **n** | **%** | **n** | **%** | **n** | **%** |
| **Yes** | 42 | 38.5 | 24 | 40.0 | 66 | 39.1 |
| **No** | 32 | 29.4 | 30 | 50.0 | 62 | 36.7 |
| **Unsure** | 35 | 32.1 | 6 | 10.0 | 41 | 24.3 |
| **TOTAL** | 109 | 100.0 | 60 | 100.0 | 169 | 100.0 |

Table S 31. Presence of strategies to harmonize multiple review processes

|  | **HICs** | | **LMICs** | | **TOTAL** | |
| --- | --- | --- | --- | --- | --- | --- |
|  | **n** | **%** | **n** | **%** | **n** | **%** |
| **Yes** | 14 | 12.7 | 12 | 20.0 | 26 | 15.3 |
| **No** | 51 | 46.4 | 41 | 68.3 | 92 | 54.1 |
| **Unsure** | 45 | 40.9 | 7 | 11.7 | 52 | 30.6 |
| **TOTAL** | 110 | 100.0 | 60 | 100.0 | 170 | 100.0 |

Table S 32. Reliance on established procedures to recognize and validate research protocol reviews conducted by other ethics committees

|  | **HICs** | | **LMICs** | | **TOTAL** | |
| --- | --- | --- | --- | --- | --- | --- |
|  | **n** | **%** | **n** | **%** | **n** | **%** |
| **Yes** | 59 | 54.6 | 38 | 63.3 | 97 | 57.7 |
| **No** | 17 | 15.7 | 18 | 30.0 | 35 | 20.8 |
| **Unsure** | 32 | 29.6 | 4 | 6.7 | 36 | 21.4 |
| **TOTAL** | 108 | 100.0 | 60 | 100.0 | 168 | 100.0 |

Table S 33. Collaboration with scientific committees that pre-reviewed or prioritized pandemic-related research protocols

|  | **HICs** | | **LMICs** | | **TOTAL** | |
| --- | --- | --- | --- | --- | --- | --- |
|  | **n** | **%** | **n** | **%** | **n** | **%** |
| **Yes** | 45 | 41.7 | 38 | 63.3 | 83 | 49.4 |
| **No** | 34 | 31.5 | 18 | 30.0 | 52 | 31.0 |
| **Unsure** | 29 | 26.9 | 4 | 6.7 | 33 | 19.6 |
| **TOTAL** | 108 | 100.0 | 60 | 100.0 | 168 | 100.0 |

Table S 34. Presence of centralized ethics review of research protocols for multicentre studies related to COVID-19

|  | **HICs** | | **LMICs** | | **TOTAL** | |
| --- | --- | --- | --- | --- | --- | --- |
|  | **n** | **%** | **n** | **%** | **n** | **%** |
| **Yes** | 51 | 46.4 | 23 | 38.3 | 74 | 43.5 |
| **No** | 29 | 26.4 | 25 | 41.7 | 54 | 31.8 |
| **Unsure** | 30 | 27.3 | 12 | 20.0 | 42 | 24.7 |
| **TOTAL** | 110 | 100.0 | 60 | 100.0 | 170 | 100.0 |

Table S 35. Formation of Joint Scientific Advisory Committees, Data Safety Review Committees, Data Access Committees, or a Joint Ethics Review Committee

|  | **HICs** | | **LMICs** | | **TOTAL** | |
| --- | --- | --- | --- | --- | --- | --- |
|  | **n** | **%** | **n** | **%** | **n** | **%** |
| **Yes** | 21 | 19.4 | 11 | 18.3 | 32 | 19.0 |
| **No** | 39 | 36.1 | 41 | 68.3 | 80 | 47.6 |
| **Unsure** | 48 | 44.4 | 8 | 13.3 | 56 | 33.3 |
| **TOTAL** | 108 | 100.0 | 60 | 100.0 | 168 | 100.0 |
